# Cultural adaptation of an evidence-based intervention to address mental health among youth affected by armed conflict in Colombia: An application of the ADAPT-ITT approach and FRAME-IS reporting protocols

**DOI:** 10.1017/gmh.2024.106

**Published:** 2024-11-28

**Authors:** María Pineros-Leano, Alethea Desrosiers, Natalia Piñeros-Leaño, Andrés Moya, Catalina Canizares-Escobar, Lyann Tam, Theresa S. Betancourt

**Affiliations:** 1 Boston College, School of Social Work, Chestnut Hill, MA, USA; 2 Brown University, Department of Psychiatry and Human Behavior, Warren Alpert Medical School, Providence, RI, USA; 3 Universidad de Los Andes, Department of Economics, Bogotá, Colombia; 4 Florida International University, Robert Stempel College of Public Health and Social Work, Miami, FL, USA; 5 Boston College, Connell School of Nursing, Chestnut Hill, MA, USA

**Keywords:** evidence-based intervention, cultural adaptation, conflict-affected youth, mental health

## Abstract

**Background:**

In Colombia, over 9 million people have been impacted by armed conflict, creating a significant need for mental health services. This study aimed to culturally adapt and pilot test the Youth Readiness Intervention (YRI), an evidence-based transdiagnostic mental health intervention, for conflict-affected Colombian youth aged 18-28 years.

**Methods:**

The eight phases of the Assessment, Decision, Administration, Production, Topical Experts, Integration, Training, and Testing (ADAPT-ITT) framework were used to culturally adapt the YRI for conflict-affected Colombian youth. The Framework for Reporting Adaptations and Modifications to Evidence-based Implementation Strategies (FRAME-IS) was used to track the adaptations made. Qualitative and quantitative data were gathered and analyzed throughout the adaptation process.

**Results:**

Data from the Assessment phase demonstrated a high need for mental health interventions among conflict-affected youth. The Testing phase revealed significant improvements in emotion regulation and functional impairment, suggesting the YRI is a promising intervention among conflict-affected Colombian youth. Qualitative data confirmed the intervention’s acceptability among youth and mental health providers.

**Conclusions:**

The YRI was successfully adapted for conflict-affected Colombian youth. Future studies using randomized designs are needed to test the effectiveness of the YRI for improving mental health among larger samples of Colombian conflict-affected youth.

## Impact statement

Despite the high rates of mental health issues in low- and middle-income countries, the use of evidence-based mental health interventions is scarce. Although Colombia has a comprehensive legal and policy framework to support conflict-affected persons, the needs of young people have not been addressed in their entirety. It is necessary to complement investments in livelihoods and education services with evidence-based mental health and psychosocial support interventions. Using the ADAPT-ITT and the FRAME-IS, the present study culturally adapted and pilot-tested an evidence-based mental health intervention for conflict-affected youth in Colombia.

## Introduction

Evidence-based mental health interventions for conflict-affected youth are sparse, despite the high rates of mental health problems and related social and economic problems in this population (Arega, 2023; Newnham et al., 2018; World Health Organization [WHO], 2017). In Colombia, over 9 million people, including 4.2 million children, adolescents, and young adults have been impacted by decades of armed conflict (Unidad de Víctimas, 2023). Youth who have been exposed to conflict experience higher rates of post-traumatic stress reactions, depression, and anxiety symptoms (Antonaccio et al., 2019; Bendavid et al., 2021; Betancourt and Khan, 2008). Adolescents who have not been directly engaged in conflict have suffered indirect effects of widespread violence, such as forced displacement, family separation, and limited access to education and health care. Many youth who have been forcibly displaced currently live in low-income neighborhoods with high levels of urban and domestic violence (Shultz et al., 2014); which further compounds the adverse effects of prior exposure to violence and loss, including higher levels of suicidal ideation, depression, hopelessness, and self-harm behaviors among youth ages 13-24 (Moya and Carter, 2019; Vahedi et al., 2023).

Although Colombia has a comprehensive legal and policy framework to support conflict-affected persons, the focus has largely been on providing basic necessities and standard socioeconomic programs, often neglecting mental health interventions tailored to the conflict and postconflict context (Fenton et al., 2024). As evidenced by previous studies in postconflict areas, whenever mental health issues are not addressed in a timely manner, youth may not be able to develop their full economic and social potential (Betancourt et al., 2012; Betancourt et al., 2014a, 2014b; Betancourt et al., 2014c; Jenkins et al., 2011; Shultz et al., 2014). Therefore, it is necessary to complement investments in livelihoods and education services provided by the Colombian government to conflict-affected youth with evidence-based mental health and psychosocial support interventions.

In recent years, The WHO has emphasized the importance of using scalable psychological interventions in communities affected by adversity, given the large disparity between the need and availability of evidence-based mental health services in these settings (WHO, 2017). Given the costs and time necessary to develop, implement, and evaluate evidence-based interventions, culturally adapting an existing evidence-based mental health intervention may accelerate implementation for new sociocultural contexts. Adaptation is defined as the deliberate process of modifying or altering the design and/or delivery of an intervention in order to increase the fit and/or effectiveness of the intervention in a given context (Wiltsey-Stirman et al., 2013). The adaptation process is vital to ensure that the needs and wants of the target population and their social and institutional contexts are considered and embedded within a given intervention (Barrera and Castro, 2006; Wiltsey-Stirman et al., 2019; Wingood and DiClemente, 2008). Despite this, modifications are often not well documented; which can hinder the systematic evaluation of their effectiveness (Barrera and Castro, 2006). Therefore, applying a clear framework for the adaptation of evidence-based interventions for new cultural contexts and populations (i.e., conflict-affected youth in Colombia) can promote the ultimate engagement and fit of the intervention, increase access to evidence-based services, and facilitate strong methodological evaluation of clinical effectiveness.

In Colombia, there have been previous efforts to address the complex needs of conflict-affected adults (Castro-Camacho et al., 2019; Castro-Camacho et al., 2023). Oftentimes, these interventions use a transdiagnostic approach in an effort to address comorbid symptoms of depression, anxiety, and posttraumatic stress (PTS), which tend to cooccur among conflict-affected populations (Mejía et al., 2024). For instance, interventions like the Unified Protocol for Transdiagnostic Treatment were successfully adopted for conflict victims in Colombia (Castro-Camacho et al., 2019), has shown significant reductions in PTS, anxiety, depression, somatic complaints, and functional impairment while enhancing quality of life, with benefits maintained three months after the intervention (Castro-Camacho et al., 2023). Despite the promising results of the Unified Protocol for Transdiagnostic Treatment, it is necessary to have an intervention focusing on the specific needs of Colombian youth.

### The present study

The current study sought to culturally adapt and pilot test an existing scalable, evidence-based mental health intervention to address the mental health needs of conflict-affected youth in Colombia. We selected the Youth Readiness Intervention (YRI; Table 1) as the intervention to be adapted. The YRI was initially developed in Sierra Leone to improve the mental health symptoms of conflict-affected youth by embedding it within schools or livelihood programs (Betancourt et al., 2012; Betancourt et al., 2014a, 2014b; Betancourt et al., 2015, 2020; Freeman et al., 2024; Newnham et al., 2015). The cultural adaptation involved a systematic process guided by the Assessment, Decisions, Administration, Production, Topical experts, Integration, Training staff, and Testing (ADAPT-ITT) framework (Wingood and DiClemente, 2008). The ADAPT-ITT framework has been used widely for cultural adaptation purposes in health and mental health research in several low- and middle-income countries and with vulnerable populations (Bass et al., 2015; Carney et al., 2020; Munro-Kramer et al., 2019). Additionally, we used the Framework for Reporting Adaptations and Modifications to Evidence-based Implementation Strategies (FRAME-IS) to track and report all adaptations made to the YRI (Miller et al., 2021). FRAME-IS categorizes the adaptations into different types (e.g., content, context, training) and levels of modifications (e.g., patient, group).

**Table 1.** Core content of the Youth Readiness Intervention (YRI)

YRI module	Main content
**Module 1:** Introductions and building group cohesion	Introduce the intervention format and goals Build motivation for group participation and cohesion among group members Elicit individualized goals for each group member Provide the definition of Specific, Measurable, Achievable, Realistic, Time Bound (SMART) goals
**Module 2:** Psychoeducation about trauma	Discuss how to monitor feelings Provide psychoeducation regarding the impact of trauma/loss on family functioning and relationships, including how trauma contributes to mood dysregulation and anger
**Module 3:** Understanding the link between beliefs, bodies, and behaviors	Discuss how beliefs, bodies, and behaviors are connected Explore healthy and unhealthy coping strategies
**Module 4:** Taking control of your life	Discuss self-care and self-regulation Introduce skills for self-regulation/mood regulation
**Module 5:** Relaxation and affect regulation	Reflect on how behaviors can alter mood Learn how progressive relaxation and guided imagery can combat tension Identify opportunities for behavioral activation
**Module 6:** Drawing on our strengths and supports to face life’s difficulties	Psychoeducation about grief and loss, and growth in the face of trauma and loss
**Module 7:** Sequential (step-by-step) problem solving & introduction to relaxation techniques	Introduce sequential problem solving via role play Application of problem-solving to participant’s SMART goals
**Module 8:** Building interpersonal skills	Explore how emotions can impact interpersonal interactions Practice positive and assertive interpersonal interactions Introduce the “Stop, Think and Speak” technique Discuss solutions to interpersonal problems
**Module 9:** Review of Coping Skills & Problem Solving	Review helpful and unhelpful coping skills Practice applying problem-solving skills Introduce and practice progressive muscle relaxation
**Module 10:** Addressing negative self-perceptions	Discuss how negative beliefs affect emotions and behaviors Facilitate identification of unhelpful or negative beliefs including hostile attributions and negative self-perceptions Introduce strategies for positive reframing/counteracting negative self-image & stigma
**Module 11:** Review of skills and relapse prevention	Review the sequential problem-solving approach Discuss and devise strategies to cope with potential future challenges Review warning signs and relapse prevention
**Module 12:** Celebration & moving forward	Reflect on skills learned throughout the intervention Reflect on relationships formed Review SMART goals and share successes Allow participants to reflect on group experience

**Source:** Adapted from *Addressing the Consequences of Violence and Adversity.* Betancourt et al., 2014c, p. 170.

## Methods

We used the eight-phase, ADAPT-ITT framework to guide our cultural adaptation process (see Table 2) and we tracked the adaptations using the FRAME-IS (Supplemental Material). Prior to initiating the adaptation process, we developed local partnerships with the High Commissioner for Peace, Victims, and Reconciliation, from the Bogotá Mayor’s Office, and a local NGO focused on peace-building. All collaborators signed a memorandum of understanding to support our collaboration on the current project. All the research activities were reviewed and approved by the Boston College Institutional Review Board (IRB) committee under protocol 22.042.01 and by the Los Andes University IRB committee under protocol 1420 of 2021.

**Table 2.** Description of the eight phases of the ADAPT-ITT framework

ADAPT-ITT Phase	Description
**A**ssessment	Conduct focus groups and a needs assessment with Colombian youth impacted by armed conflict Conduct key informant interviews with key stakeholders (e.g., youth, parents, community leaders, service providers) Analyze results of formative evaluations
**D**ecision	Decide which intervention to adapt based on evidence and similarity of context Decide on initial adaptations based on review of needs assessment findings Produce the first draft of the culturally adapted manual
**A**daptation	Administer the theater test of intervention with Colombian youth impacted by conflict and obtain feedback Review theater test feedback
**P**roduction	Produce a second draft of the culturally adapted manual
**T**opical Experts	Engage topical experts (e.g., disciplinary experts, local culture, and language experts) Topical experts review session content and provide feedback
**I**ntegration	Integrate feedback from topical experts into the third draft of the culturally adapted manual
**T**raining	Train intervention facilitators Obtain feedback from facilitators Produce the fourth draft of the culturally adapted manual
**T**esting	Prepilot test the adapted intervention Administer pre- and postintervention assessments Analyze quantitative and qualitative prepilot findings Make any further revisions to the culturally adapted manual Produce the fifth draft of the culturally adapted manual

### Phase 1: assessment

**Recruitment:** In collaboration with the High Commissioner for Peace, Victims, and Reconciliation, we used a two-pronged approach to recruit participants residing in the neighborhood of Bosa, an area in the Southwest of Bogotá with a high proportion of forcibly displaced persons, from September 30 through November 12, 2021. Participants were recruited to take part either in focus group discussions or semistructured key informant interviews. Eligibility for focus group discussion participants was: (a) aged 18-25; (b) self-reported that they had been impacted by armed conflict; and (c) residing in Bosa. The initial age range was selected to target youth, following the definition by the United Nations (2004) and the initial age range used by the YRI in Sierra Leone (Betancourt et al., 2014a, 2014b). Eligibility for key informant interviews was: (a) aged 18 and over; (b) lived within Bosa, and (c) were either: community leaders, providers employed by the Mayor’s Office, or conflict-affected people.

Members of the High Commissioner for Peace and Reconciliation generated a list and contacted 168 potential participants in coordination with study research assistants (RAs) to explain the purpose of the study and invite them to participate in a focus group. All potential participants were registered in the *Registro Unico de Victimas*, an official registry that identifies victims of the armed conflict. From the potential participants who answered the phone and agreed to participate in the study, 24 attended the day the focus groups were scheduled. The focus groups were held in community centers within Bosa.

The second recruitment approach consisted of working with community leaders in the Bosa neighborhood in order to enter community spaces where youth and other community leaders congregated. RAs attended community meetings to describe the purpose of the study and invite youth and community leaders to participate in the interviews. From these meetings and through referrals from community leaders, 25 potential participants were identified, and two other participants were recruited via snowball sampling.

A recurrent challenge throughout the study was that it was difficult to recruit male participants. Innovative recruitment strategies were used to address the lack of male engagement, including creating spaces to play soccer prior to the focus groups. Despite participation in soccer games, male participants withdrew from discussions when focus groups started.

**Procedure:** We conducted a total of six focus group discussions and 32 key informant interviews. Focus groups and interviews focused on identifying the needs of the community, determining their priorities, and identifying potential barriers and facilitators to participating in an evidence-based intervention developed specifically for youth impacted by armed conflict in Colombia (See Supplemental Material). Focus group discussions were audio-recorded and lasted an average of 90 minutes. Participants received a snack during the focus group discussion and $2 USD for transportation. Participants provided written consent at the community centers before the focus groups began. Key informant interviews were held either in person or via phone. All interviews were audio-recorded and lasted approximately 25 minutes. Semistructured interview guides included questions about the types of activities and jobs that youth aged 18-25 engaged in and the types of family responsibilities they held. All focus group discussions and interviews were transcribed verbatim in Spanish. Participants provided written or verbal consent before the interviews began. All participants filled out a short demographic survey that included information about gender, age, time spent living in Bogotá, educational level, and job description.

**Qualitative data analysis**: Qualitative data analysis was conducted by two bilingual and bicultural RAs trained in qualitative analysis methodology. Qualitative data were coded in Spanish using a multistage thematic network analysis approach (Attride-Stirling, 2001). As part of the first stage, the RAs read all the transcripts and assigned generic codes using Dedoose Version 9.0.17 (Dedoose, 2021). In the second stage, the RAs worked with one of the principal investigators (MPL) to develop a codebook. Both RAs then coded all the transcripts using the developed codebook and identified illustrative quotes for each code. Once all data (i.e., key informant interviews and focus groups) had been coded, the RAs developed a thematic network where codes were grouped into categories, which were then grouped into larger themes (Creswell and Poth, 2016). Selected quotes from participants were translated from Spanish to English using translation and back-translation (WHO, n.d). Intercoder reliability agreement was determined by comparing the consistency with which each coder applied specific codes in the interviews. To calculate this, the number of interviews in which both coders applied the same code was identified, and the agreement between coders was measured. This was done by dividing the number of agreements by the total number of coding decisions made for that code, providing a percentage that reflects the level of agreement between coders. Each code had to have at least an 80% intercoder agreement. Whenever a code did not reach this threshold, the coders, and the study investigator met to discuss the definition of the code until general consensus was reached. The RAs then recorded the interviews with less than 80% agreement. The final intercoder reliability agreement for this study was 86%.

### Phase 2: decision

**Procedure:** Given the lack of mental health interventions tailored for conflict-affected youth in Colombia, the YRI, which has shown effectiveness in improving interpersonal functioning, emotion regulation, and mental health among conflict-affected youth in Sierra Leone (Newnham et al., 2015; Betancourt et al., 2014 and Freeman et al., 2024, see Table 3), was selected as the intervention that would be adapted to the Colombian context. The YRI was selected given that it was initially developed in a postconflict setting, similar to the postconflict Colombian context. Moreover, the YRI was originally developed as a transdiagnostic intervention to address comorbid mental health issues and improve functional outcomes among youth in both school and livelihood settings, making it the ideal intervention for our target population.

**Table 3.** Scientific evidence of the youth readiness intervention in Sierra Leone

Study	Sample and design	Results
YRI Pilot trial (Newnham et al., 2015)	Pre-post-test design with a sample of 32 conflict-affected youth in Sierra Leone ages 15–24 years.	The results suggested that the YRI was acceptable and feasible. The results indicated a reduction in internalizing and externalizing symptoms, improved daily functioning, and emotion regulation, with an average effect size of *d = 0.64.*
YRI Randomized Controlled Trial (Betancourt et al., 2014)	Pre, post-, 6-month, and 8-month follow-up design with a sample of 436 conflict-affected youth in Sierra Leone ages 15–24 years.	The results indicated that after the intervention, prosocial behaviors, emotion regulation, and social support improved. Additionally, the study showed a decrease in school dropouts and an improvement in behavior within the classrooms.
YRI with Entrepreneurship Training (ENTR) (Freeman et al., 2024)	Cluster randomized design. Clusters were randomly assigned to one of three conditions: 1) YRI+ENTR, 2) ENTR, and 3) Control. Data were collected at baseline, midline ((6–7 weeks after baseline), and at endline (8–9 weeks after baseline). The sample consisted of 1,151 conflict-affected youth in Sierra Leone ages 18–30 years.	The results showed that youth who received the YRI and ENTR showed significantly greater improvements in mental health, daily functioning, and labor market outcomes compared to youth who only received ENTR and compared to control youth.

Results from the Assessment phase suggested that the YRI was a good fit for conflict-affected youth and potentially successful integration into an entrepreneurship program as tested previously in other conflict-affected settings to help youth develop skills such as emotion regulation, goal-setting, and communication as well as provide a shared space to talk and listen to their peers.

The YRI was originally developed in Sierra Leone in partnership with community members. The original intervention was developed by mental health researchers and experts using information from key informant interviews, focus groups with former child soldiers, community leaders, parents, and police officers from Sierra Leone (Betancourt et al., 2014a; Zuilkowski et al., 2016). The YRI initially consisted of 10 modules that incorporated various therapeutic techniques such as cognitive restructuring, behavioral activation, self-regulation, monitoring, relaxation skills, psychoeducation on trauma, and interpersonal skills (Betancourt et al., 2014a). However, a modification was made to the initial YRI, adding two more modules to include emotion monitoring and grief/loss to improve the ability of the intervention to address anxiety and depression, following its early trial in education settings (Betancourt et al., 2014a). This final intervention, with a total of 12 modules, was the version we adapted in this process (See Table 1).

### Phase 3: adaptation

**Recruitment:** We conducted eight theater test modules between November 26 and December 5, 2021, with separate groups for males and females. We recruited and enrolled 12 male participants and 25 female participants from the communities in Bosa. The theater test modules were conducted within community spaces, close to where participants lived, and ranged in duration between 49 and 105 minutes, with a median of 90 minutes.

Theater testing is a methodology where participants attend the demonstration of an intervention (Wingood and DiClemente, 2008). During the theater tests, facilitators implemented modules of the program, and at the end of each module, participants and observing stakeholders responded to questions about the core elements, materials, delivery style, and activities within the modules (Wingood and DiClemente, 2008). The feedback helped determine the appropriateness of the content and suggested what might be added or removed to better fit the target population. Following this phase, facilitators, participants, and stakeholders discussed how to incorporate the feedback to refine the intervention, ensuring it meets the needs and preferences of the intended audience (Wingood and DiClemente, 2008).

**Procedure:** Eight (out of 12) modules from the YRI were selected to be tested using theater tests. Two modules were excluded because they focused on the review of material from previous modules and the other two modules were excluded due to time constraints. Three RAs were trained on the core content of the YRI modules by study investigators. Attendance to the theater tests varied, with male groups having at least five males attending, and the female group having at least 12 females attending per module. Participants received a snack during the theater test and $2 USD for transportation. During the theater test module, the participants received the entire module and then participated in a semistructured interview with facilitators to better understand what participants liked about the module, what they did not like, and what they thought would improve the module (See Supplemental Material). The RAs took notes of the participants’ comments, which were then shared and discussed with the principal investigators and informed of the core adaptations of the YRI manual.

### Phase 4: production

**Procedure:** In this phase, the research team combined and analyzed all the data collected in the adaptation phase to produce the first version of a culturally adapted manual for Colombian youth impacted by conflict.

### Phases 5 and 6: topical experts and integration

**Procedure:** Two topical experts were selected to provide feedback on the culturally adapted YRI manual. One topical expert was selected given the strong research and practice experience he has working with victims of armed conflict in Colombia. The topical expert conducted a thorough revision of the cultural aspects of the adapted manual and provided feedback related to each module. The feedback from the expert was documented in the manual itself, and then, a Word document with a summary of the main recommendations was shared with the research team. The second expert was the primary intervention developer and an expert on cross-cultural mental health research. The intervention expert reviewed the main changes that had been made to the intervention and provided further feedback. The feedback from the intervention expert was documented in a Word document, which was then shared with the research team. After both experts provided feedback, the research team met and discussed the recommendations from the experts and decided on the specific changes that would be implemented, which are reflected in Table 4. Feedback from the experts was focused on the cultural content of the manual, the format, and the length of the intervention, as well as ensuring that the core content was retained. Based on the feedback provided by the two topical experts and the discussion from the research team, a second version of the manual was developed with additional adaptations.

**Table 4.** Modifications made to the YRI by module and by phase of the ADAPT-ITT framework

Overall changes	Modifications	ADAPT-ITT Phase
**Delivery style and Logistics**	Providing childcare was essential to ensure that participants could fully engage and benefit from the modules. Women favored women-only groups, while men generally preferred mixed-gender groups. Gender-specific groups were maintained in order to provide safe spaces to disclose difficult topics. The titles of all the modules were revised and changed to more meaningful titles in Spanish.	Adaptation
**All modules**	Used gender-inclusive terminology throughout the manual.	Topical experts and integration
	Two review modules were removed from the manual to improve clarity and avoid redundancy, reducing the total number of modules to 10.	Training, Topical experts, and integration
	The word ¨Homework¨ was changed to ¨Challenge of the day¨ to promote adherence.	Training
**Intervention name**	Jovenes Capibara	Training
**Appendix**	Added Icebreakers and fun activities as optional resources in the appendix of the manual.	Training
**Module 1: Introductions and Building Group Cohesion**	An activity with body movement was added as an ice-breaker in the introduction.	Adaptation
	The activity of the “mystic” was added. In this activity, during each module at the beginning, an object was placed in the center of the circle by each participant and it had to have a meaning for them. At the end of the module, the object was retrieved and had gathered the participants’ energy. Added pronouns to the introduction, along with an explanation of what they meant and why they are important. Added information on diversity and inclusion, its importance, and the importance of respecting cultural, racial, intellectual, and sexual diversities.	Testing
**Module 2: Trauma Psychoeducation**	Module 2 includes an analogy of a pot of boiling water, which is a tool that helps participants become aware of their emotions and monitor them. Through this analogy, participants can monitor how the water gradually warms up and if it is not monitored, it may spill everywhere. This analogy was replaced with a pot of boiling chocolate, which is very common in Colombia and consumed on a daily basis in many households. Examples of traumatic experiences were changed to be more contextual and to something that participants could relate to. For example, the invasion in Freetown was mentioned in this module and it was changed to difficult situations that IDPs might have gone through, such as receiving threats, being subject to forced displacement, sexual violence, and forced disappearance, among other victimizing events in the Colombian context.	Adaptation
	The introduction to trauma symptoms, education about the effects of trauma, re-experiencing, and avoidance in the trauma context was moved to the fifth module as it was advised by one of the topical experts that this content came too early in the intervention and more trust had to be built before starting this discussion. The word ¨trauma¨ was changed to ¨psychosocial damage¨, as advised by the providers from the Mayor’s Office. Diaphragmatic breathing was initially a skill taught in module four, but it was moved up to module two.	Training
**Module 3: Understanding the Link Between Beliefs, Bodies, and Behaviors**	The order of the second and third modules was switched given that facilitators suggested it was necessary to understand the relationship between thoughts, emotions, and behaviors, before moving forward with more complex topics.	Testing
**Module 4: Taking Control of Your Life**	Information related to being a “watchman” and monitoring one’s emotions was taken out given that the message was not properly conveyed. A whole new section on Mindfulness was included. A definition of mindfulness, along with activities to understand and practice Mindfulness was added.	Adaptation and training
**Module 5: Relaxation and Affect regulation**	Introduction to trauma symptoms, education about the effects of trauma, re-experiencing, and avoidance, which were initially part of module 2, were moved to module 5. This change was made because addressing trauma so early in the intervention felt premature. By the fifth module, participants are more likely to have established relationships and feel comfortable sharing and discussing this topic. Additionally, the “Safe Place” imagery skill introduced in module 5 can help participants feel secure and prepared to engage with the trauma-related content.	Adaptation
**Module 6: Drawing on our strengths and supports to face life’s difficulties**	Progressive muscle relaxation, which was part of module 9, was added to this module.	Adaptation
**Module 7: Sequential (step-by-step) Problem Solving & Introduction to Relaxation Techniques**	No major changes were made to this module.	
**Module 8: Building Interpersonal Skills**	No major changes were made to this module.	
**Module 9: Review of Coping Skills & Problem Solving**	This module was eliminated since it was a review module.	Training
**Module 10: Addressing Negative Self-Perceptions**	No major changes were made to this module	
**Module 11: Review of Skills and Relapse Prevention**	This module was eliminated since it was a review module.	Training
**Module 12: Celebration & Moving Forward**	An activity to review all the skills learned throughout the intervention was added to this module.	Adaptation
	An activity from module 11 preparing youth for future challenges and avoiding relapse was moved to this module.	Training

### Phase 7: training

**Procedure:** We recruited 16 community members (aged 18 – 43 years; female = 7; male = 9) to participate in a two-week, in-person training of the culturally adapted manual. Additionally, three providers from the Mayor’s Office (female = 2, male = 1) participated and provided daily feedback about each module, suggesting additional changes based on their extensive experience working with internally displaced populations (IDPs). The training was conducted in Spanish by one of the principal investigators (MPL; a native Spanish speaker). Facilitators were selected based on their interest in learning more about the intervention and their experience as community leaders within their neighborhoods. Daily feedback modules were held with RAs and representatives from the High Commissioner who had observed the training to gain input on additional recommendations for modifications to the manual. This feedback was then incorporated into a final revision of the manual.

### Phase 8: testing

**Recruitment:** We recruited and enrolled 28 participants in the testing phase. Inclusion criteria were youth: (a) aged 18-28 and (b) self-reported impact by armed conflict. The pilot program was expanded to include youth aged 18 to 28 years old for two primary reasons: 1) The Colombian Statutory Law 1885 of 2018, defines “youth” as “any person between 14 and 28 years of age,” (Congreso de la República, 2018) and 2) both providers and participants recommended broadening the age range to reach a larger and more diverse population. RAs called potential participants identified in the list from the High Commissioner to describe the purpose of the pilot phase and invite them to participate. Participants who had been part of previous phases and expressed their interest to continue participating were also eligible.

**Procedure:** Between February 23, and March 20, 2022, the culturally adapted intervention was piloted using the fourth version of the culturally adapted manual. A total of five community youth (female = 4; male = 1) and one male RA who had been trained in the previous phase were selected to implement the intervention. In this phase, a total of 10 modules were delivered to three groups; two groups of females and one group of males (N = 28; female group A n = 15, female group B n = 7, and male group n = 9). The groups met three times per week for two hours. During each meeting, the facilitators delivered one module, for a total of three modules per week delivered to each group. The pacing of the intervention was based on the availability of each group’s participants. Qualitative and quantitative data were collected during the testing phase of the study. Quantitative data were collected at baseline and postintervention. Qualitative data were collected via exit interviews (N = 6) (see Supplemental Material) and one focus group (N = 7) with youth from the testing phase, and also from previous phases (e.g., theater testing phase). Quantitative and qualitative data were collected by RAs who were present during the implementation of the intervention.

**Measures:** All quantitative scales were administered in Spanish by trained study RAs. All study measures were previously validated in Colombia (Bohórquez-Borda et al., 2023; Camargo et al., 2021; Cassiani-Miranda et al., 2021; Paniagua, 2017; Üstün et al., 2010) and demonstrated strong psychometric properties in the current study during baseline (α = 0.87 - 0.96; Table 5) and postintervention (α = 0.73 - 0.94; Table 5).

**Table 5.** Psychometric properties of scales used with Colombian adolescents

Scale	Number of items	Cronbach’s Alpha baseline	Cronbach’s Alpha postintervention
PHQ–9	9	0.87	0.73
GAD–7	7	0.89	0.90
DERS-SF	18	0.89	0.92
WHODAS	39	0.96	0.94

Symptoms of depression were collected via the Spanish version of the Patient Health Questionnaire-9 (PHQ-9). Anxiety symptoms were collected via the Spanish version of the General Anxiety Disorder Schedule (GAD-7). Emotion regulation was assessed using the Difficulties in Emotion Regulation-Short Form (DERS-SF). Functional impairment was assessed using the World Health Organization Disability Assessment Schedule (WHODAS), which consists of 39 items scored on a Likert scale.

**Quantitative data analysis:** Data from the quantitative scales were analyzed using Stata 16 following the guidelines from each individual scale. For the baseline and postintervention assessments, continuous scores were constructed to identify the severity of symptoms of depression, anxiety, difficulties in emotional regulation, and functional impairment. In addition, dichotomous measures indicating levels of symptoms indicative of a likely clinical disorder were estimated for anxiety and depression following the thresholds proposed by each scale (Bohórquez-Borda et al., 2023; Camargo et al., 2021; Cassiani-Miranda et al., 2021; Paniagua, 2017; Üstün et al., 2010). Because of the small sample size, we estimated differences in means pre- and postassessment in both the continuous scores and whether or not youths scored in the likely clinical range.

**Qualitative data analysis:** Qualitative data analysis for the Testing phase followed the same procedures as those described under the Assessment phase. The average intercoder reliability agreement for the Testing phase was 97.6%.

**Overall decisions to modify the intervention:** During all phases that included recommendations for adding, tailoring, substituting, or removing any components of the intervention, the researchers—including Principal Investigators, Co-Investigators, RAs, and the developer of the intervention—gathered at different moments and held meetings to discuss whether each suggested change should be implemented. This decision-making process was based on their collective experience with the population and the intervention. Most of the suggested modifications were applied, as they did not involve major changes to the core content of the intervention (see Table 4 for more details about the section removed). Changes made during Phase 8 were also discussed and changes were made to the final version of the culturally adapted manual for future iterations of the intervention.

## Results

### Phase 1: assessment

**Focus Group Discussions:** A total of 40 youth participated in six focus groups (2 male-only groups, three female-only, and one mixed-gender). The number of participants per group ranged from four to nine, and the average age was 21.8 years (SD = 2.2; Table 6). The majority of participants (n = 21; 52.5%) were students.

**Table 6.** Demographic information from focus group participants

Focus group	Sex	Number of participants	Age range	Mean	Standard deviation
1	Female	7	18–24	21.4	1.9
2	Female	4	23–25	24.0	0.8
3	Female	9	18–25	21.3	2.7
4	Mixed	6	21–25	22.6	1.5
5	Male	5	22–25	23.0	1.2
6	Male	9	18–24	20.4	2.5

**Key Informant Interviews**: 32 participants were part of the individual interviews. Most participants were community leaders (n = 19; 59.4%) and identified as female (n = 20; 62.5%). The average age was 41.5 years (SD = 14.5). Eleven interview participants (43%) had less than a high school degree.

Findings from the focus groups and key informant interviews revealed four themes related to the issues that conflict-affected youth faced in their community: 1) Lack of opportunities 2) Family mistrust, 3) Behavioral health issues related to difficult circumstances, 4) Gender-specific challenges, and 5) Social and structural supports for youth.

***Lack of opportunities:*** Participants mentioned that while many youth in their community pursue a high school education, the journey to higher education remains arduous. Some participants indicated that only a select few manage to enroll in institutions like “SENA” (Servicio Nacional de Aprendizaje), a Colombian public institution that provides vocational training to students. Moreover, the majority of participants mentioned that there were limited opportunities for employment after completing high school, leaving young people without opportunities to engage with school or employment (Table 7 includes sample quotes from participants).

**Table 7.** Sample quotes from participants from the assessment phase

Theme	Sample quotes
Lack of Opportunities	“…because here there is a lack of opportunities for the youth… many do nothing. I mean, many do not occupy their minds with anything, they do not study, they do not work, [they do] nothing at home, so there is also the risk of that, these are youths who have all the time to think about other things that they should not do.” Mother, interview, age 39 “No, here they do not even help for sports or those skills that young people have and that is what makes the lack of opportunities, that young people tend to go down other paths which are frowned upon by society, but there is nothing else to do, right? Because if you do not have time to dedicate to studying, to work, or to dedicate yourself to a sport that you like or something, then you are going to dedicate yourself only to being on the street, I say this because of my classmates at school, there were 45 of us when we graduated from high school, only 10 of us were able to access higher education, I am talking about SENA or university, and the rest dedicated themselves to wander around..” Female youth, female focus group, employee, age 21
Family Mistrust	“Aaaa yes, it is like you answer when they ask you, are you okay? and you say yes I am fine, to avoid uncomfortable answers. But that is why you repress how you feel by avoiding that or they are not the person you want to express what you feel and sometimes these things happen, it is easier to express everything you feel to the person you just meeting, to the person I am seeing their face for the first time than to your own family, to people who live with you. Because sometimes they are very intolerant, like oh you again with that thing and you and your sentimentalism.” Female youth, female focus group, unemployed, age 24 “And that’s why you also see so many cases of children already like oh, my dad isn’t paying attention, so let’s try this or do this, ehh no, but it is just that my mom isn’t there, so I’m going to go to a friend´s house, and my mother is not going to say anything. Also, if I come home, I´m going to be alone. Oooh no, but my mother rewards me for everything, so since they are so absent, the only thing they do is reward the children, that is, reward them and reward them, and that generates many difficulties even when people are young. And then they begin to see all the changes and say oh no my daughter became more rebellious. But why is the child in that? So like everything has a mechanism, everything starts happening that way, but it’s also because everything comes from a very young age.” Female youth, female focus group, student, age 18 “But it turns out that there are mothers who see a child in that situation and instead of advising them, of trying to bring them in, of having a little patience with them, we tell them to “go away!” You are useless, you do nothing. Or also cajole what they bring home, those are also mistakes of us parents for me.” Mother, interview, age 58
Behavioral Health Issues Related to Difficult Circumstances	“I feel that there is a strong lack of support for young people when you search unless you [already] have the services, it is not the norm, because what happens is that you go to them, and in the process, they tell you to fill out these forms and you make the appointment and then they make you wait and I find these cases all the time… what I feel with all those authorizations is distrust…” Youth, mixed-gender focus group “Being young is tough because not all of us have the possibility to just study, because it’s difficult for us, sometimes we have responsibilities like contribute to the household, or work or study, or I can’t handle both things, so I say that many times young people dedicate themselves only to work and don’t have the opportunity to study because they don’t have the support of a family member, something to support them, well, now in my case, I’ve been fortunate because my siblings have always been supporting me because if it weren’t for them, I wouldn’t be here, because I don’t have a job, I don’t have anything, and thanks to the support they give me and my family, I can study and I’m at home. I say that most young people kind of go through that, I mean they do not have the opportunity for that, I mean like work, help at home, or have problems with family so they have an emotional problems and sometimes they leave home early and do not have aspirations to study or move forward or anything because they say this was the life I was dealt and I think it is like they end up on the wrong path like in drug addiction, yes, that was my contribution” Female youth, female focus group, student, age 24
Gender-specific challenges	“Well, the truth is that as a woman you don’t feel safe anywhere. Whether I am in Bosa or Suba [two different locations in Bogota] or anywhere else, I do not feel safe. As a woman, you do not feel safe. I am a very alert person, although I am riding in Transmilenio [public transport] and there I have met girls who do not even realize that a man is doing “that” [masturbating] and I always have it in mind because I am always on the lookout…” Female youth, female focus group, student, age 21 “Yes, but relatively things have a huge impact, like when… I have to come by bus sometimes, and I had not come here to see my mom for about 20 days because I had taken the T38, a bus service, and the man leaned back behind me, and behind, and behind, and behind. I had not noticed, I arrived here, normal, and my mom said, ‘What do you have there?’ And of course… well, I had all of this full of… ” Female youth, female focus group, employee, age 25
Social and Structural Supports for Youth	“I’m sure that if you structured a project, everyone here would approve of it, right? So now you are the teachers: “Tomorrow we meet, tomorrow we are going to do such work, we are going to dig”, you set up a bunch of leather goods like making straps, and shoes, tomorrow we start the factory, you are going to work, to learn… If you have your mind busy all day, you don’t have time to think about things, right? But if you are unemployed, you think, my girlfriend left me, my mother does not love me…” Female participant, interview, community leader, security personnel, age 40 "The truth is, just as we are here, first they listen to us and then through each one’s needs, one starts looking at what program fits, just like that. For example, let’s suppose what each one likes to do. Let’s say I like cooking, doing a cooking day. Do you understand me? It is like activities to keep the young people from being so discouraged. So they’re not on the streets so they do not pick up bad habits or anything like that” Female youth, interview, street vendor, age 22

***Family mistrust:*** The youth also mentioned they faced issues with their family members. Some participants indicated that their parents were often too busy working to spend time with them, which made them feel like their parents did not care about them. Participants also mentioned feeling pressured by their parents to either pursue higher education and/or find a job to contribute to the household financially. The majority of participants indicated that this ongoing pressure from parents, compounded with the lack of opportunities, created a situation in which adults perceived youth as irresponsible and lacking a strong work ethic, and youth viewed adults with mistrust, making it difficult to openly communicate with them.

***Behavioral health issues related to their circumstances:*** Another theme that arose was that many young people struggled with symptoms of depression and suicidal ideation. Participants mentioned that given the lack of available opportunities, they sometimes became withdrawn, isolated, and in some cases discussed thoughts of suicide with their peers. Some participants indicated that these feelings emanated from the lack of support from the adults in their lives. Participants also mentioned that although there might be mental health services available specifically for IDPs, these services were not accessed due to long waitlists and a general distrust of government institutions. Youth participants stated that they felt like the government did not care about them and their wellbeing, and cared only about adding a number to their caseload to claim that they were serving the population.

***Gender-specific challenges***: In the female focus groups, many talked about the high prevalence and normalization of sexual harassment. Participants mentioned feeling extremely vulnerable when using public transportation and feeling scared when walking alone in their neighborhoods for fear of being sexually harassed. Female participants reported very difficult situations that they experienced and the traumatic sequelae of some of these experiences. Female participants also discussed the additional difficulties of finding a job because some opportunities were only available to men. Finally, participants discussed difficulties faced by female youth who are also parents, particularly with respect to balancing work, school, and childcare needs.

***Social and structural supports for youth***: A final theme mentioned by participants was the desire to help other youth succeed. Participants mentioned providing more spaces where youth could engage in recreational activities and also engage with adults from the community in an effort to bridge the generational gap and strengthen relationships between youth and adults. Participants also mentioned providing more support and guidance to youth through the provision of behavioral health services that were not affiliated with government entities. Finally, the participants suggested providing more opportunities for youth to develop specific skills (i.e., vocational or technical skills) or where they could learn how to become entrepreneurs.

### Phase 2: decision

Given the similar context where the YRI was developed, its target population, and its extensive research evidence, the research team selected the YRI as the intervention to be culturally adapted to the Colombian context (see Table 1 with the YRI’s core components).

### Phase 3: adaptation

Throughout the theater tests, youth were highly involved and showed to be willing and interest in participating in the intervention. Participation fluctuated throughout the eight modules. To ensure full engagement, groups were capped at 15 participants, with additional people recruited to account for potential no-shows. In the female theater test group (n=25; μ age = 22.96 years), out of 25 participants who were recruited to participate, only nine participants attended the first module (See Supplemental Material). In the male theater test group (n=12; μ age = 21.16 years), out of 12 recruited participants, seven attended the first module.

### Phase 4: production

Based on feedback obtained from the youth who participated in the theater tests, we made three main modifications to the intervention. First, we changed the focus from trauma and grief that resulted from the exposure to armed conflict to trauma and grief related to more general events, such as daily life and urban violence. This change was made given that most participants in the theater tests stated that many of them had arrived in Bogotá when they were very young and did not have direct experience with victimizing events from the armed conflict. Additionally, the youth mentioned that in their neighborhoods, there is daily exposure to other events, which they can relate to and are open to discussing. Some of these events of urban violence were added to the manual as examples. Second, the opening activity for each module was developed between one of the RAs and one of the theater test groups. This activity consisted of identifying a physical space in the room where each participant placed an object that had sentimental value to them with the purpose of gathering the energy of each person present in the space. The participants indicated that this opening activity should be included as an opening and closing activity throughout the duration of the intervention. The final change consisted of reducing the number of modules from 12 to 10. In order to achieve this, activities from modules 9 and 11 were added to modules 6 and 12 (see Table 4). We also included culturally relevant icebreakers that would increase the discussion of feelings and emotions to help participants trust the facilitators and normalize discussions around these topics. Finally, we included activities with more physical movement in outdoor areas since the youth indicated they spent too much time sitting and would prefer being outdoors when possible.

### Phases 5 and 6: topical experts and integration

Following the feedback provided by the Colombian expert, we ensured that the language used throughout the manual was inclusive of male and female genders, creating a more gender-inclusive manual. Additionally, in the original manual, there were sporadic religious references (e.g., proverbs) and activities that were removed, given that Colombia is a secular state. Another change consisted of expanding the discussion around grief to include examples of possible events to which Colombian youth could be exposed in their daily life. These comments were incorporated into the third version of the culturally adapted manual to increase the appropriateness to the Colombian context while retaining core intervention components.

### Phase 7: training

In this phase, additional adaptations were made based on the recommendation from the providers and future facilitators. The first adaptation consisted of moving content on trauma psychoeducation and normalizing reactions to extreme events from the second to the fifth module. This content was moved down to module five given that by then the group had built rapport and felt more comfortable discussing trauma-related topics, making it a more suitable point in the intervention. A second adaptation included the removal of the concept of the “Watchmen/Watchwomen” because it would not culturally translate. The intended purpose of the “Watchmen/Watchwomen” is to promote self-efficacy by closely monitoring one’s emotions. However, this concept was continuously interpreted as “the voice of conscience” and it was always accompanied with judgment and self-criticism. Despite varied efforts to convey the concept of self-efficacy, participants continued to act self-critically. Given that the intended message was not properly conveyed in Spanish and with the population we were working with, we decided to remove this concept. Since self-criticism was a common practice among our population, we focused on reinforcing the concept of mindfulness. Additionally, some light-touch changes suggested included adding the definitions of terms and definitions are also added to the manual to ensure that everyone has a similar starting point regarding issues like emotion, emotion regulation, trauma, psychosocial damage, and mindfulness. Also, following recommendations from facilitators, more activities and more information on how to properly carry out the closing module were also included in the manual.

One last step in the cultural adaptation process was to name the culturally adapted intervention. We conducted a brainstorming module where everyone suggested different names, which were written on a whiteboard. All suggestions were subjected to a vote and the name with the highest number of votes was selected. The chosen name for the intervention was Jóvenes Capibara (Capybara Youth) because the Capybara animal is one of the most social animals, and it allows other animals to sit or rest on their back. This name resonated with the youth because they felt that the program helped them support each other through the different tools learned in the program. These suggested changes were incorporated into the manual, generating a fourth version.

### Phase 8: testing

**Quantitative results**: At baseline, 26% of participants presented a score above PHQ-9 and the GAD-7 cut-offs, respectively suggesting at least mild symptoms of depression and anxiety (Table 8). At the postintervention assessment, symptoms of depression were 1.75 points lower than in the pre-intervention assessment, corresponding to a 27% reduction. Regarding anxiety symptoms, these were 2.16 points lower, corresponding to a 34% reduction; emotion regulation difficulties were 7.60 points lower, corresponding to a 31% decrease; and functional impairment was 11.78 point lower, corresponding to an 18% decrease. Although all symptoms showed reductions from baseline to postintervention, statistically significant changes were only observed in the scores for emotion regulation difficulties (DERS) and functional impairment (WHODAS). In addition, anxiety risk levels decreased from 26% at baseline to 14% at postintervention, and depression risk levels decreased from 26% at baseline to 10% at postintervention, which corresponds to 46 and 61 percent reductions relative to baseline scores. However, these changes did not reach statistical significance, most likely because of the small sample size.

**Table 8.** Pre- and postintervention scores and at-risk symptoms

Measure	Pre-intervention	Post-intervention	Difference
	(1)	(2)	(2–1)
PHQ–9 Score	6.52	4.76	–1.75
	(6.01)	(5.19)	[1.564]
PHQ–9 Risk cut-off (=1)	0.26	0.14	–0.12
	(0.45)	(0.36)	[0.112]
GAD–7 Score	6.35	4.19	–2.16
	(5.51)	(4.46)	[1.387]
GAD–9 Risk cut-off (=1)	0.26	0.1	–0.16
	(0.45)	(0.30)	[0.103]
DERS Score	24.74	17.14	–7.60^**^
	(13.01)	(11.78)	[3.473]
WHODAS Score	65.45	53.67	–11.78^**^
	(23.69)	(17.02)	[5.647]
Observations	31	21	52

Note:

** *p* value < 0.05.

**Qualitative results:** The following four themes were identified: 1) Positive experiences throughout the intervention, 2) Skills/abilities learned by participants, 3) Barriers to participation, and 4) Suggestions to improve the intervention.

***Positive experiences throughout the intervention:*** Nearly all participants reported positive experiences and outcomes throughout the program. Youth underscored the importance of support from other participants and the community that was developed through the program (see Table 9 for sample quotes). Participants also highlighted that the facilitators created an environment that supported individual growth. Participants reported feeling loved, understood, and validated within the group, which allowed them to vent their emotions and experiences comfortably. Participants also mentioned that through the program they were able to achieve a greater sense of self-confidence, motivation, and ability to establish goals.

**Table 9.** Sample quotes from participants from the testing phase

Theme	Sample quotes
Positive Experiences Throughout the Intervention	“The dynamics, sharing with all of you, learning about other people, realizing that I am not the only one with problems or difficulties, that there are more people who also need that support”. Female youth, homemaker, age 22 “The compatibility is what provided relief. It was about being relaxed and sharing with people who understand.” Female youth, various jobs, age 22 “My satisfaction was at its peak, not only because of you who were the tutors of this whole experience, but also because of my companions, who made the experience even more motivating.” Female youth, student, age 20
Skills/Abilities Learned During Participation	“Another aspect that I think was talked about a lot is the ability to first think, breathe, enjoy the wind, and then act, or first organize my ideas and not just talk for the sake of talking.” Female youth, student, age 25 “For instance, I taught my brother everything they taught me there; I shared it with him. Sometimes he has his temper, and I would tell him, ‘Do not do that. You have to, when you get angry, breathe, do not be rude.” Female youth, homemaker, age 22 “Indeed, one is left in awe. There are women who make you think, ‘Wow.’ It is also inspiring because you hear stories that make you realize—it is not about mocking, but about recognizing that there are women who experience sorrow and yet also find joy. It makes you appreciate, ‘How inspiring.’ It is enlightening to learn all this. It also encourages us to assert, ‘Women, you have the power.” Female youth, self-employed, age 24
Barriers to Participation	“No, ma’am. It’s just that I couldn’t come back because at that time I was working, and the schedules didn’t allow me the possibility to be involved in the project and work.” Female youth, various jobs, age 22 “The second time was because my daughter got sick because really, one says, ‘Oh no,’ she had such a strong cough that kept me awake all night and I had to rush for that. It’s not the same as having mindfulness, which is like we were talking about there, one is thinking about something else and not paying attention.” Female youth, independent contractor, age 24 “Yes, I think I couldn’t attend maybe two sessions precisely because of that, the issue of timing is very difficult because I work and that’s why I have a somewhat heavy schedule at university because I study in the afternoon-evening and also study on Saturdays.” Female youth, student, age 25
Suggestions to improve the intervention	“That says a lot, but unfortunately, I would have liked to have more time. I think it is no longer a problem with the program, but a very personal issue. As I said, perhaps it could have been a little more extensive.” Female youth, student, age 25 “Yes, I feel that there is a lack of security in the first sessions, like being able to study the agenda in advance, arriving more confident, more open, not just staying with the booklet because that makes me feel very anxious, I do not know how to handle very extreme levels of anxiety. It is like saying, ‘If you do not know what we are going to do, then what are we going to do?’. Yes, more expression toward the center, toward everyone, but I feel that in the end it was achieved.” Female youth, independent contractor, age 23 “More opportunities for groups like this. Because just once isn’t enough, something more frequent, more constant.” Female youth, various jobs, age 22

***Skills/Abilities learned during participation*:** The majority of participants reflected on the various topics addressed during the intervention. Notably, several participants provided detailed accounts of the elements of the intervention that had a profound and lasting impact on their lives. For instance, they highlighted the acquisition of positive communication skills and coping mechanisms, such as the ‘stop-think-act’ model, alongside breathing techniques. These tools improved emotion regulation and bolstered self-confidence when navigating challenging situations. The majority of participants indicated that the abilities they learned helped them foster a greater sense of empathy and heightened their social and problem-solving skills. Participants also indicated that they liked the skills they learned so much that they often shared with family members, relatives, and friends what they had learned during the intervention (Desrosiers et al., 2021). Participants also emphasized the program’s effectiveness in strengthening their resilience, particularly by providing a safe environment where they could openly share their challenging experiences and successful coping strategies. These shared stories were instrumental in redefining their personal approaches to difficulties and enhancing their sense of empowerment.

***Barriers to participation***: A third main theme related to the barriers experienced by participants to access the program. Participants mentioned schedule conflicts and a shortage of support available to supervise their children while they were attending intervention modules. Participants also mentioned a lack of personal motivation to complete the program and a lack of support from others to follow through and attend the modules regularly.

As mentioned earlier, male participation was a challenge that remained throughout the study. For example, after the intervention, only four male participants agreed to participate in the exit interviews, but none of them showed up to their scheduled interview regardless of several efforts made by the RAs to contact them.

***Suggestions to improve the intervention***: The final theme involved suggestions made by participants to improve future iterations of the intervention. Several participants highlighted that although they really liked that facilitators were peers from their communities, they felt they needed more in-depth training before facilitating the intervention. Participants also suggested including more modules to deepen community connections and skills learned.

## Discussion

This study conducted a systematic cultural adaptation of an evidence-based mental intervention initially developed to address the mental health needs of youth impacted by armed conflict in West Africa to the context of Colombian youth impacted by armed conflict (Bernal and Domenech Rodríguez, 2012). Guided by community-based participatory research methods and using the ADAPT-ITT framework, we worked alongside community members and leaders to ensure that their everyday realities were reflected in the cultural adaptation of the intervention and to ensure that the intervention responded to their daily struggles. Findings provide preliminary support for the need for culturally appropriate, evidence-based mental health programs for IDP youth in Colombia as well as for the feasibility, acceptability, and potential benefits of a peer-delivery model of Jovenes Capibara. One important aspect of the culturally adapted intervention is that it follows a peer-delivery model, where other community youth, without formal mental health training, deliver the modules. Previous studies have demonstrated that peer-delivery models are effective mental health delivery models in low-resource settings (Freeman et al., 2023; Pfeiffer et al., 2011; Singla et al., 2017). However, to ensure the safety of participants, it is necessary to develop strong risk-of-harm protocols. For the present study, given the partnership with the Mayor’s Office, we had two psychologists who were readily available to talk to participants in case they felt the need to receive professional mental health support. Also, there were referral protocols where participants were referred to a mental health professional if they expressed suicidal or homicidal ideation.

Overall, results from the testing phase showed significant improvements in emotion regulation and functional impairment, indicating the intervention is promising among Colombian youth impacted by armed conflict. Qualitative results also indicated that the intervention was acceptable among Colombian youth and community-trained facilitators. However, youth indicated that in order to improve intervention adherence, it was necessary to couple the intervention with a skill-building or entrepreneurship program, similar to what has been done in Sierra Leone and other contexts. Versions of mental health programs coupled with employment or skill-building programs have been successfully implemented in similar contexts, including Sierra Leone (Betancourt et al., 2020; Desrosiers et al., 2023; Freeman et al., 2024), India (Balaji et al., 2011), and Uganda (Ssewamala et al., 2012). Following the recommendations by participants and based on principles from the original intervention, future iterations of Jovenes Capibara should be integrated within an entrepreneurship program.

### Strengths and limitations

This study has important strengths. First, although the community and the local government understand the mental health issues of youth impacted by armed conflict, and/or the urban violence to which they are exposed upon arrival, they do not have a strong infrastructure with a wide array of programs and services that can effectively help reduce mental health issues within this specific population. Therefore, the cultural adaptation of this intervention was relevant and timely. Second, the use of a clear framework such as ADAPT-ITT allowed the intervention to be culturally appropriate and acceptable to participants.

This study has a few limitations worth mentioning. The sample size of the testing phase (N = 29) was rather small, and it is possible that null findings in depression and anxiety were a result of this. A second limitation is that the pilot was carried out only within one neighborhood in Bogotá. Although Bogotá is diverse and attracts people from various regions of Colombia, our sample did not fully capture this diversity. Moreover, we did not include a question about the race of our participants, making it difficult to determine whether our sample included participants who identified as indigenous or Afro-Colombian. Engaging male participants was a challenge from the beginning and was a recurring problem throughout all stages. This challenge differed from studies conducted in Sierra Leone, where males comprised 50% or more of the sample (Newnham et al., 2015; Betancourt et al., 2014b; Freeman et al., 2024). It is possible that cultural factors play a role, as it may be seen as inappropriate for males to openly discuss their emotions, especially in male-dominated spaces (bell hooks). Another possibility is that the pacing of the intervention was too fast, not allowing enough time to internalize and process the skills learned during the modules. Future iterations of the intervention should be delivered no more than twice per week. Future research should focus specifically on the barriers to recruitment and retention of young Latin American males in mental health and psychosocial support programs.

## Conclusions

Using the ADAPT-ITT framework, we followed eight steps to adapt an evidence-based intervention, the YRI, to address the mental health concerns of conflict-affected Colombian youth. The cultural adaptation process aimed to ensure that the adapted intervention was acceptable among conflict-affected Colombian IDPs living in Bogotá. Initial findings suggested improvements in emotion regulation and functional impairment among conflict-affected Colombian youth. However, it is necessary to use a rigorous randomized controlled trial design to evaluate effectiveness in addressing mental health among conflict-affected youth living in Colombia using a robust design going forward. Moreover, future studies should test the intervention in different contexts and with a more diverse youth population to ensure its feasibility and acceptability.

## Supporting information

Pineros-Leano et al. supplementary material 1 Pineros-Leano et al. supplementary material

Pineros-Leano et al. supplementary material 2 Pineros-Leano et al. supplementary material

Pineros-Leano et al. supplementary material 3 Pineros-Leano et al. supplementary material

Pineros-Leano et al. supplementary material 4 Pineros-Leano et al. supplementary material

Pineros-Leano et al. supplementary material 5 Pineros-Leano et al. supplementary material

## Data Availability

The dataset, which includes individual transcripts, is not publicly available due to confidentiality policies.

## Supplementary material

The supplementary material for this article can be found at http://doi.org/10.1017/gmh.2024.106.
