# Supplementary material for: Cultural adaptation of an evidence-based intervention to address mental health among youth affected by armed conflict in Colombia: An application of the ADAPT-ITT approach and FRAME-IS reporting protocols
Source: Glob Ment Health (Camb). 2024 Nov 28;11:e114. doi: 10.1017/gmh.2024.106 (PMC11704387; doi:10.1017/gmh.2024.106)
Supplement: Pineros-Leano et al. supplementary material 1 — Pineros-Leano et al. supplementary material [file S2054425124001067sup001.docx]

Participant attendance in the Adaptation phase by session

| Female Theatre Test Group (N = 25) | Male Theatre Test Group (N = 12) |
| --- | --- |
| YRI Module 1: 9 participants | YRI Module 1: 7 participants |
| YRI Module 2: 9 participants | YRI Module 2: 7 participants |
| YRI Module 4: 7 participants | YRI Module 4: 9 participants |
| YRI Module 6: 9 participants | YRI Module 6: 9 participants |
| YRI Module 7: 12 participants | YRI Module 7: 7 participants |
| YRI Module 8: 10 participants | YRI Module 8: 5 participants |
| YRI Module 10: 7 participants | YRI Module 10: 5 participants |
| YRI Module 12: 7 participants | YRI Module 12: 5 participants |
