# Supplementary material for: Cultural adaptation of an evidence-based intervention to address mental health among youth affected by armed conflict in Colombia: An application of the ADAPT-ITT approach and FRAME-IS reporting protocols
Source: Glob Ment Health (Camb). 2024 Nov 28;11:e114. doi: 10.1017/gmh.2024.106 (PMC11704387; doi:10.1017/gmh.2024.106)
Supplement: Pineros-Leano et al. supplementary material 2 — Pineros-Leano et al. supplementary material [file S2054425124001067sup002.docx]

**Exit Interview Question Guide**

1. Overall, how have you been doing since participating in Jovenes Capibara?

2. What was it like for you to participate in the program?

What were the things you liked or didn't like about the program?

How would you describe the experience of being on the program?

3. How likely were you to participate in the intervention?

What challenges, if any, did you face when participating in the intervention?

4. How likely were you to try to find time to repeat some activities and strategies learned through the program?

How satisfied were you with the time between sessions?

Was it enough to help you practice what you learned during the program?

What did you learn in the program that you shared with your friends or family?

5. Overall, how satisfied were you with the program?

Was there anything that would have gone very well on the program?

Was there anything that didn't go so well with the program?

6. How did participating in the program impact you? Includes examples.

What changes have you noticed in your family since the program ended?

How did things stay the same?

Is there any particular difficulty you are currently facing?

7. What has changed in the way you interact with your friends and family afterwards ypur participation in Jovenes Capibara?

8. Was there anything new you learned during the program that you didn't know before?

9. How was it working with your facilitator? What was it like being in a group with other young people from your community?

What did you like or dislike about your facilitator? Include examples.

In what way did you feel satisfied or dissatisfied with your facilitator?

Was there anything that your facilitator did well?

Was there anything that your facilitator DIDN'T do very well?

10. How, if anything, can we improve the program to help youth in Colombia whose families have been displaced?

11. Is there anything I haven't asked you that you would like to share? Please feel free to let me know any additional thoughts or ideas you have related to the program.
