# Supplementary material for: Cultural adaptation of an evidence-based intervention to address mental health among youth affected by armed conflict in Colombia: An application of the ADAPT-ITT approach and FRAME-IS reporting protocols
Source: Glob Ment Health (Camb). 2024 Nov 28;11:e114. doi: 10.1017/gmh.2024.106 (PMC11704387; doi:10.1017/gmh.2024.106)
Supplement: Pineros-Leano et al. supplementary material 3 — Pineros-Leano et al. supplementary material [file S2054425124001067sup003.docx]

**Interview and Focus Group Question Guide**

1. Please tell me more about the young people between the ages of 18 and 24 in your community. What kind of activities do they do? What kind of jobs do they have? If any What family and/or household responsibilities do they have?
2. What kind of problems, if any, affect young people between the ages of 18 and 24 in your community?
3. If yes, what kind of difficulties with emotional well-being affect young people? (Emotional well-being refers to the ability of young people to talk about emotions and connect socially and with the world around us. Social well-being includes such things as the social skills people have, the way they cope with problems, and the ability they have to feel and manage different emotions). [Researchers: provide this information to participants, and then provide the space for them to define what emotional well-being means to them.]
4. Can you give an example of a young person who may have this type of problem with emotional well-being (don't use anyone's real name)?
5. Please tell me more about the type of problem this person experienced.
6. What do you think may have caused the problem?
7. When young people have these kinds of problems, who helps them? What do they do to get help?
8. What types of services are available in the community to help with these types of problems?
9. Please tell me in what other ways can young people handle problems like these?
10. What is the community doing to help young people who have these problems? Please give an example.
11. What is the community doing that can contribute to solving these problems they may have? Please give an example.
12. Please tell me about other ways that people in the community can help young people with problems like these.
13. If you were designing a program to help youth with these types of problems, what would you do? What things would be important to consider in designing such a program to help youth in the community?
14. Now we would like you to help us choose a name for the Program. We are thinking about [provide options].
