# Supplementary material for: Cultural adaptation of an evidence-based intervention to address mental health among youth affected by armed conflict in Colombia: An application of the ADAPT-ITT approach and FRAME-IS reporting protocols
Source: Glob Ment Health (Camb). 2024 Nov 28;11:e114. doi: 10.1017/gmh.2024.106 (PMC11704387; doi:10.1017/gmh.2024.106)
Supplement: Pineros-Leano et al. supplementary material 4 — Pineros-Leano et al. supplementary material [file S2054425124001067sup004.docx]

|  |  |
| --- | --- |
| **FRAME-IS module or sub- component** |  |
| **Module 1** |  |
| The EBP being implemented is: | Youth Readiness Intervention (YRI) |
| The implementation strategy being modified is: | The manual |
| The modifications being made are: | Tailoring language in the manual to fit Colombian Youth context |
|  | Adding, substituting elements |
|  | Removing elements |
|  | Re-ordering elements |
|  | Spreading elements |
| The reasons for the modifications are: | Improve appropriateness and acceptability |
| **Module 2** |  |
| What is modified? | Context (Setting, based on transition from Sierra Leone to Colombia) |
|  | Content |
| **Module 3** |  |
| What is the nature of the content modification? | Tailoring language in the manual to fit Colombian Youth context |
|  | Adding and substituting elements |
|  | Removing elements |
|  | Re-ordering elements |
|  | Spreading elements |
| **Module 4** |  |
| What is the goal? | Increase the acceptability, appropriateness, and feasibility of the program/manual |
| What is the level of the rationale for the modification? | Provider and recipient level |
| **Module 5** |  |
| When is the modification initiated? | Pre-implementation |
| Is the modification planned? | Planned/proactive |
| **Module 6** |  |
| Who participates in the decision to modify? | Researcher, program coordinators, facilitators who were trained and topic expert |
| Who makes the ultimate decision? | Researchers |
| **Module 7** |  |
| How widespread is the modification? | Provider/facilitator level |
|  | Population level |
