# Supplementary material for: Cultural adaptation of an evidence-based intervention to address mental health among youth affected by armed conflict in Colombia: An application of the ADAPT-ITT approach and FRAME-IS reporting protocols
Source: Glob Ment Health (Camb). 2024 Nov 28;11:e114. doi: 10.1017/gmh.2024.106 (PMC11704387; doi:10.1017/gmh.2024.106)
Supplement: Pineros-Leano et al. supplementary material 5 — Pineros-Leano et al. supplementary material [file S2054425124001067sup005.docx]

**Theatre Test Methodology**

1. In general, what did you think about the session?
2. Considering that there are between 10 and 12 sessions, how often would you like to receive these sessions? (e.g. once a week, twice a week?)
3. Where would you prefer to receive these sessions or this type of information? (for example, hand in hand with another program, if yes, what type of program? Like another program from the Bogotá Mayor's Office or the High Commissioner for Peace, Victims and Reconciliation?
4. Information about victimizing events/emotional duels: At your age, what are the emotional struggles that young people deal with the most? (e.g. breakup, domestic violence)
5. Would you feel more comfortable having sessions only with people of the same gender? What do you think might be some of the problems with having mixed sessions?
6. If we do separate sessions. What do you think would be comfortable for a person who is part of the LGBTQ community?
7. Which person would do you think should implement/facilitate the sessions? (e.g. community workers, community adults, community leaders, friends).
