# Supplementary material for: Cultural adaptation of an evidence-based intervention to address mental health among youth affected by armed conflict in Colombia: An application of the ADAPT-ITT approach and FRAME-IS reporting protocols
Source: Glob Ment Health (Camb). 2024 Nov 28;11:e114. doi: 10.1017/gmh.2024.106 (PMC11704387; doi:10.1017/gmh.2024.106)
Supplement: Peer review history

# Editor report: Recommendation: Cultural adaptation of an evidence-based intervention to address mental health among youth affected by armed conflict in Colombia: An application of the ADAPT-ITT approach and FRAME-IS reporting protocols — R0/PR2

**DOI:** 10.1017/gmh.2024.106.pr2

**Affiliations:** Ponce Health Sciences University, Puerto Rico

No accompanying comment.

# Editor report: Decision: Cultural adaptation of an evidence-based intervention to address mental health among youth affected by armed conflict in Colombia: An application of the ADAPT-ITT approach and FRAME-IS reporting protocols — R0/PR3

**DOI:** 10.1017/gmh.2024.106.pr3

**Affiliations:** London School of Hygiene & Tropical Medicine, United Kingdom of Great Britain and Northern Ireland

No accompanying comment.

# Editor report: Recommendation: Cultural adaptation of an evidence-based intervention to address mental health among youth affected by armed conflict in Colombia: An application of the ADAPT-ITT approach and FRAME-IS reporting protocols — R1/PR5

**DOI:** 10.1017/gmh.2024.106.pr5

**Affiliations:** Ponce Health Sciences University, Puerto Rico

No accompanying comment.

# Editor report: Decision: Cultural adaptation of an evidence-based intervention to address mental health among youth affected by armed conflict in Colombia: An application of the ADAPT-ITT approach and FRAME-IS reporting protocols — R1/PR6

**DOI:** 10.1017/gmh.2024.106.pr6

**Affiliations:** London School of Hygiene & Tropical Medicine, United Kingdom of Great Britain and Northern Ireland

No accompanying comment.
